# Compressive Behavior of (Cu_0.47_Zr_0.45_Al_0.08_)_98_Dy_2_ Bulk Metallic Glass at Different Strain Rates

**DOI:** 10.3390/ma13245828

**Published:** 2020-12-21

**Authors:** Yu-Ting Wang, Xu-Dong Zu, Xiang-Kui Liu, Zheng-Xiang Huang, Peng-Gang Jin, Jian Kong

**Affiliations:** 1 School of Mechanical Engineering, Nanjing University of Science and Technology, Nanjing 210094, China; wangyuting@njust.edu.cn (Y.-T.W.); huangyu@njust.edu.cn (Z.-X.H.); 2 School of Material Science and Engineering, Nanjing University of Science and Technology, Nanjing 210094, China; xkliu93@163.com (X.-K.L.); kongjian68@126.com (J.K.); 3 Xi’an Modern Chemistry Research Institute, Xi’an 710065, China; jinpenggang204@126.com

**Keywords:** bulk metallic glass, mechanical properties, fracture behavior, shear fracture, normal fracture

## Abstract

The mechanical properties of (Cu_0.47_Zr_0.45_Al_0.08_)_98_Dy_2_ bulk metallic glass (BMG) were characterized under various strain rates by quasi-static and dynamic compressive tests. In the quasi-static compressive tests, the yield stress of (Cu_0.47_Zr_0.45_Al_0.08_)_98_Dy_2_ BMG increased from 1234 MPa to 1844 MPa when the strain rate was increased from 0.001 s^−1^ to 0.01 s^−1^, and the yield stress decreased to 1430 MPa at the strain rate of 0.1 s^−1^. In the dynamic compressive tests, when the strain rate increased from 1550 s^−1^ to 2990 s^−1^, the yield stress of (Cu_0.47_Zr_0.45_Al_0.08_)_98_Dy_2_ BMG first decreased from 1508 MPa to 1404 MPa, and then increased to 1593 MPa. The fracture behaviors of (Cu_0.47_Zr_0.45_Al_0.08_)_98_Dy_2_ BMG were studied by using scanning electron microscopy to examine the fracture surface. Fracture occurred in the pure shear mode with strain rates below 2100 s^−1^, whereas shear fracture and normal fracture occurred simultaneously under strain rates of 2650 s^−1^ and 2990 s^−1^.

## 1. Introduction

Since the discovery of bulk metallic glasses (BMGs), they have been receiving attention from researchers because of their excellent properties, such as high strength and a high corrosion resistance [1,2,3,4,5]. In particular, Cu-based BMGs have been the focus of research due to their high glass-forming capacity and thermal stability [6,7,8,9,10,11]. Cu-based BMGs also have the advantages of the easy availability of raw materials and low cost. Therefore, they have enormous potential for wide applications in engineering. 

Recently, researchers have begun to investigate the application potential of BMGs in the field of impact dynamics [12,13] The mechanical behavior of BMGs with different compositions under different strain rates were studied. Bruck [14] and Lu [15] found that at strain rates of 10^2^ s^−1^ to 10^3^ s^−1^, the strain rate effect of Zr_41.25_Ti_13.75_Cu_12.5_Ni_10_Be_22.5_ BMG is insignificant at room temperature. Li [16] found that with the increase of strain rate, the strain rate effect of Zr_52.5_Cu_17.9_Ni_14.6_Al_10_Ti_5_ BMG first showed insensitive and then exhibited negative sensitivity. The strain rate effect of Zr/Hf-based [17] and Pd-based BMGs [18] is negatively sensitive. The yield stress of Zr_53_Cu_30_Ni_9_Al_8_ [19], Ti_45_Zr_16_Ni_9_Cu_10_Be_20_ [20], and Nd_60_Fe_20_Co_10_Al_10_ [21] BMGs increase along with the strain rate. Hsu [22] found that the plastic flow stress value of Ti-Cu-Ni-Al_x_ bulk metallic glasses increased with the increases of strain rate. The current research on the mechanical properties of Cu-based BMGs is mainly concentrated in the low-strain-rate range [23,24]. Thus, to tap the application potential of Cu-based BMGs applied in dynamic events, their mechanical behavior at different strain rates should be investigated.

This research characterized the quasi-static compressive behavior and dynamic compressive behavior of (Cu_0.47_Zr_0.45_Al_0.08_)_98_Dy_2_ BMG by using a DNS100 universal testing machine (Sinotest Equipment Co., Ltd., Changchun, China) and Split Hopkinson pressure bar (SHPB) (Nanjing university of science and technology, Nanjing, China), respectively. To investigate the fracture mechanisms, the fracture surface of the specimens after tests was observed by scanning electron microscopy (SEM) (FEI Quanta 250F scanning electron microscope, Portland, OR, USA). The data of this study can provide a reference for the application of (Cu_0.47_Zr_0.45_Al_0.08_)_98_Dy_2_ BMG.

## 2. Materials and Experimental Program

In this paper, the raw materials were high-purity Cu (99.95 mass%), Zr (99.99 mass%) Al (99.99 mass%), Dy (99.9 mass%). Under argon atmosphere (purity of 99.999%), (Cu_0.47_Zr_0.45_Al_0.08_)_98_Dy_2_ ingots were prepared in the arc-melting furnace (Sky technology development Co., Ltd. Shenyang, China) according to atomic percentage of nominal compositions. These ingots were repeatedly smelted and flipped at least 3–5 times to ensure the homogeneity of the alloy composition. The measurement of the metal ingots that completed the arc-smelting indicated that their mass deviation was less than ± 0.01%. Subsequently, the rods with diameter of 5 mm and 3 mm were made by copper mold ejection casting technique at high vacuum under the pressure of 0.05 MPa. The crystalline structure of (Cu_0.47_Zr_0.45_Al_0.08_)_98_Dy_2_ BMG was measured by X-ray diffraction (XRD) (Bruker D8 X-ray diffractometry with Cu Kα radiation, Ettlingen, Germany). The specimens with diameter of 3 mm and height of 6 mm were prepared for quasi-static compressive tests, and the specimens with diameter of 5 mm and height of 3 mm were prepared for dynamic compression tests and XRD measure.

The specimens were sanded with 1000, 2000, 3000 times sandpaper, and then fully polished with a polishing liquid with a particle size of 0.3 μm for 10 min. The surface of the specimen was observed by scanning electron microscope, and no flaws were found. The detection results of the laser scanning confocal microscope showed that the surface roughness of all specimens was about 0.2 μm. The surface states of all specimens tended to be the same, thus, the experimental results were not affected by the surface states of the specimens.

Quasi-static uniaxial compressive tests of (Cu_0.47_Zr_0.45_Al_0.08_)_98_Dy_2_ BMG were conducted at strain rates of 0.001, 0.01, and 0.1 s^−1^ on the DNS100 universal testing machine (Sinotest Equipment Co., Ltd., Changchun, China).

**Figure 1:**
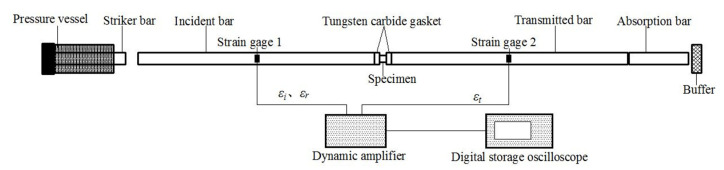
Schematic of the SHPB test.

Dynamic uniaxial compressive tests of (Cu_0.47_Zr_0.45_Al_0.08_)_98_Dy_2_ BMG were performed using SHPB. Figure 1 shows the schematic of the SHPB apparatus, where the lengths of the striker bar, incident bar, and transmitted bar are 300, 1500, and 1500 mm, respectively. The strain gauge 1 on the incident bar surface measures incident and reflected signals. The strain gauge 2 on the transmitted bar surface measures the transmitted signals.

Usually, the stress $\sigma_{s}$, strain $\varepsilon_{s}$, and strain rate $\frac{d\varepsilon_{s}}{dt}$ in the specimen can be calculated as follows:

$$\left\{ \begin{array}{l} \sigma_{s}=\frac{EA}{A_{s}}\varepsilon_{t}\\ \varepsilon_{s}=\;-\frac{2c_{0}}{l_{0}}\int_{0}^{t}\varepsilon_{r}dt\\ \frac{d\varepsilon_{s}}{dt}=-\frac{2c_{0}}{l_{0}}\varepsilon_{r} \end{array}\right.$$

where E, A, and $c_{0}$ refer to the elasticity modulus, cross sectional area, and elastic wave velocity of pressure bars, respectively. $A_{s}$ and $l_{0}$ are the cross-sectional area and length of the specimen, respectively. $\varepsilon_{t}$ and $\varepsilon_{r}$ are the reflected signals and transmitted signals measured by strain gauge in the test, respectively. 

To increase the rise time of the ramp incident pulse to be equivalent to the time required for the stress to equilibrate within the specimen, so that the data obtained in tests is valid, a square brass sheet was placed between the incident bar and the striker bar to generate a ramp pulse [25,26,27]. The SHPB test device was improved by a tungsten carbide gasket whose resistance was matched with the pressure bar.

## 3. Results and Discussion

### 3.1. Structure Analysis of (Cu_0.47_Zr_0.45_Al_0.08_)_98_Dy_2_ BMG

**Figure 2:**
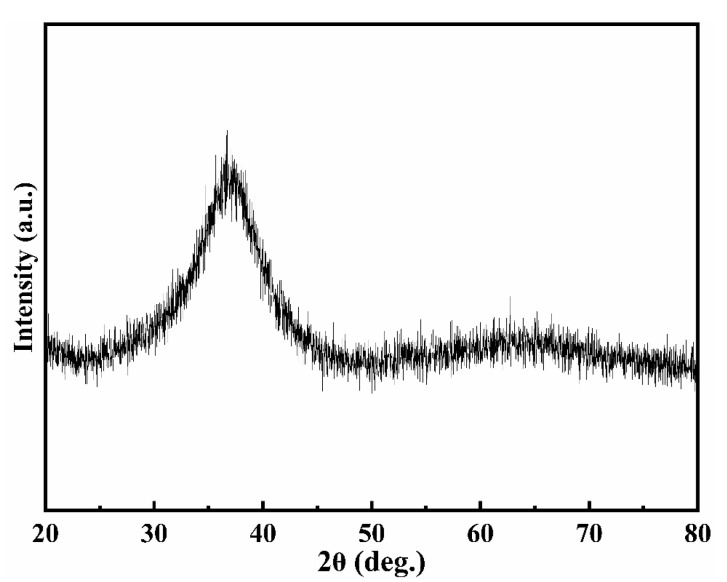
XRD pattern of (Cu_0.47_Zr_0.45_Al_0.08_)_98_Dy_2_ BMG.

Figure 2 shows the XRD pattern of (Cu_0.47_Zr_0.45_Al_0.08_)_98_Dy_2_ BMG. Only one broadened and diffused hump was observed under diffraction in the 2θ range, and no sharp peaks corresponding to the crystalline phase were observed, which indicated that the specimens were completely amorphous.

### 3.2. Mechanical Properties

**Figure 3:**
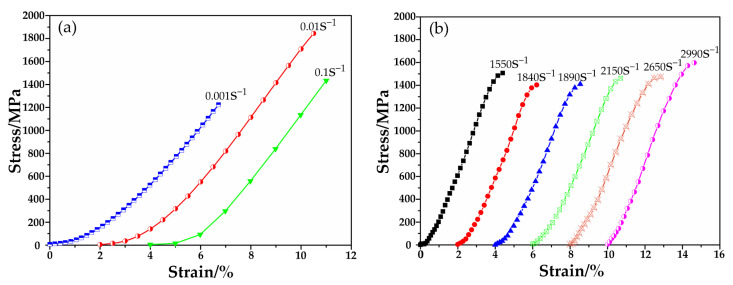
Stress-strain relations of specimens at different strain rates. (**a**) Quasi-static compressive tests and (**b**) dynamic compressive tests.

Figure 3 shows the stress–strain relations of (Cu_0.47_Zr_0.45_Al_0.08_)_98_Dy_2_ BMG at quasi-static and dynamic compressive tests at room temperature. In order to clearly show multiple curves in the figure, each curve was displaced by a 2% strain. 

In the dynamic tests, to ensure that the (Cu_0.47_Zr_0.45_Al_0.08_)_98_Dy_2_ BMG specimen was in an equilibrium state of stress and that the failure occurred at the uniform strain rate, a square brass sheet of different sizes was used to adjust the incident waveform. The incident waveform, especially its rising edge, showed obvious differences under different strain rates [13]. Thus, the “elastic range” of the dynamic stress-strain curves showed a difference.

**Figure 4:**
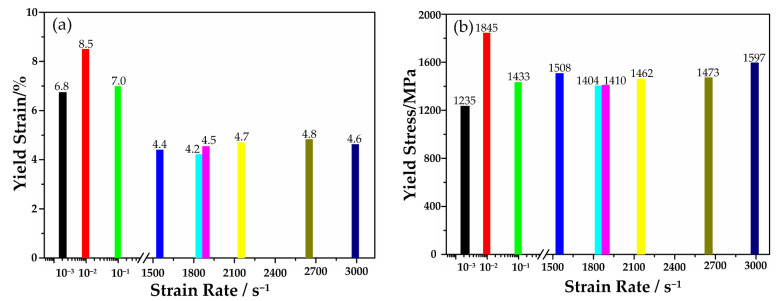
(**a**) Yield strain and (**b**) yield stress of the involved compressive tests.

Figure 4a shows that the yield strain of the quasi-static compressive tests was obviously larger than that of the dynamic compressive tests, which may be attributed to two reasons. One reason could be that the specimen undergoes different stresses in the quasi-static and dynamic compressive tests. With the low loading rate in the quasi-static compressive tests, the radial expansion rate of the specimen caused by Poisson’s effect can be ignored. The specimen was mainly subjected to axial compressive stress, and shear band propagation was constrained. Thus, a large amount of elastic strain could be accumulated. In the dynamic compressive tests, the specimen underwent high-rate deformation, and the specimen expanded radially and generated kinetic energy in the lateral direction because of Poisson’s effect [13]. Therefore, in addition to axial compressive stress, the specimen was also subjected to radial tensile stress, and shear band propagation was less constrained [18]. Thus, the accumulated elastic strain during the deformation was smaller. The other reason could be the size difference of the specimen in quasi-static and dynamic compressive tests. The energy of material deformation stored before the fracture was mostly dissipated as heat [28]. Wu [29] indicated that a smaller specimen diameter corresponded to a smaller energy dissipation density, resulting in a more stable shear band. Thus, more elastic strains could be accumulated before the macroscopic fracture.

Similar to most BMGs, (Cu_0.47_Zr_0.45_Al_0.08_)_98_Dy_2_ BMGs exhibited brittle behavior, and no macroscopic plastic deformation was observed. The fracture of (Cu_0.47_Zr_0.45_Al_0.08_)_98_Dy_2_ BMGs could be regarded as the nucleation and propagation of microcracks, leading to macroscopic failure [30]. At the microscopic level, the initiation of the shear band could be regarded as the threshold for microcrack nucleation [15]. 

Figure 4b shows the yield stress of (Cu_0__.47_Zr_0.45_Al_0.08_)_98_Dy_2_ BMGs at different strain rates. In the quasi-static compressive tests, when the strain rate increased from 0.001 s^−1^ to 0.01 s^−1^, the yield stress increased significantly, as the similar trends for traditional brittle metals and ceramics [31]. This finding could be attributed to inertial effects limiting the rate of crack growth [17,32]. While the strain rate increased to 0.1 s^−1^, the yield stress decreased. This was probably because additional shear bands at other potential nucleation sites would be initiated, and their growth would be inhibited at higher compressive strain rates [16]. In the dynamic compressive tests, the specimen underwent radial tensile stress due to the radial expansion of the specimen caused by Poisson’s effect, and fracture occurred rapidly as soon as the shear band began to propagate [18]. Liu [33] indicated that the number of the shear bands initiated at dynamic tests was higher than that at quasi-static tests. The higher the strain rate, the more shear bands initiated. Thus, the yield stress of (Cu_0.47_Zr_0.45_Al_0.08_)_98_Dy_2_ BMG decreases at strain rates below 1890 s^−1^. The reason for the positive strain rate sensitivity in the range of 2100 s^−1^ to 2990 s^−1^ needs further discussion.

### 3.3. Fracture Behavior

**Figure 5:**
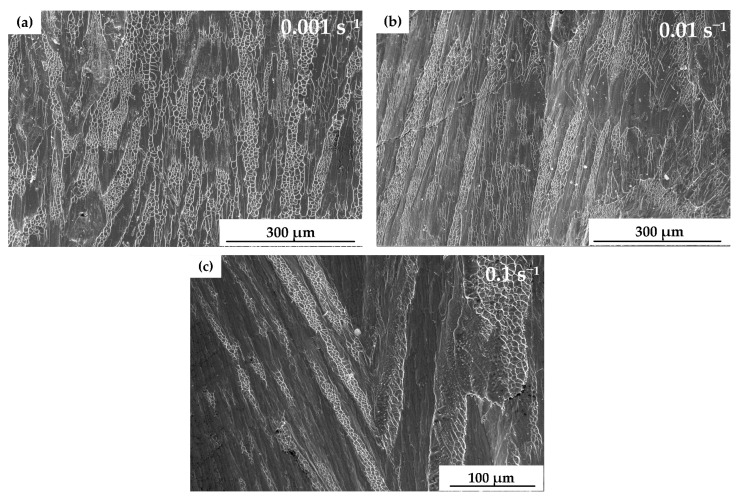
SEM micrographs of the fracture surface of quasi-static compressive tests. (**a**) 0.001 s^−1^, (**b**) 0.01 s^−1^, and (**c**) 0.1 s^−1^.

Figure 5 shows the SEM images of the fracture surface of (Cu_0.47_Zr_0.45_Al_0.08_)_98_Dy_2_ BMG during the quasi-static compressive tests. The fracture surface exhibited a typical compressive shear fracture: a vein-like pattern. BMGs are free of the local inhomogeneous structures seen in crystalline metallic materials, such as grain boundaries, twins, and dislocations [34]. Moreover, their deformations exhibit shear localization until finally catastrophic failure at room temperature [15]. Due to this deformation mechanism, the deformation is constantly accumulated in the shear bands, and the shear bands continue to expand in an unstable way, resulting in a vein-like pattern [23,31,35].

At the strain rate of 0.001 s^−1^, the shear band propagated slowly, thus, although the distribution directions of the vein-like patterns were roughly along one direction, there was also interlacing between the vein-like patterns. With an increase of strain rate, such as 0.01 s^−1^ and 0.1 s^−1^, the propagation velocity of the shear band increased. Thus, the vein-like patterns were clearly distributed along one direction. Figure 5 indicates that fracture under quasi-static compressive tests occurred in the pure shear mode.

Near the vein-like pattern and the crack propagation area, molten droplets could be observed. The reason for this could be that a large amount of elastic energy stored in the deformation process was released within the highly localized shear bands. The temperatures in the shear bands increase rapidly. First, the temperature rises to the glass transition temperature of the material, the structural changes and the melt-like behavior occurs [36]. Then, the temperature rises to close to, or even over, the melting point of the material, and the material melts [35,37].

The specimen during dynamic compressive tests underwent rapid deformation. The huge elastic energy stored during the deformation was released in a short time, which rapidly heated up the specimen, causing the partially broken specimen to melt and adhere to the tungsten carbide gasket. Therefore, the overall fracture morphology could not be observed. The local fracture morphology was selected to investigate its fracture mechanism.

**Figure 6:**
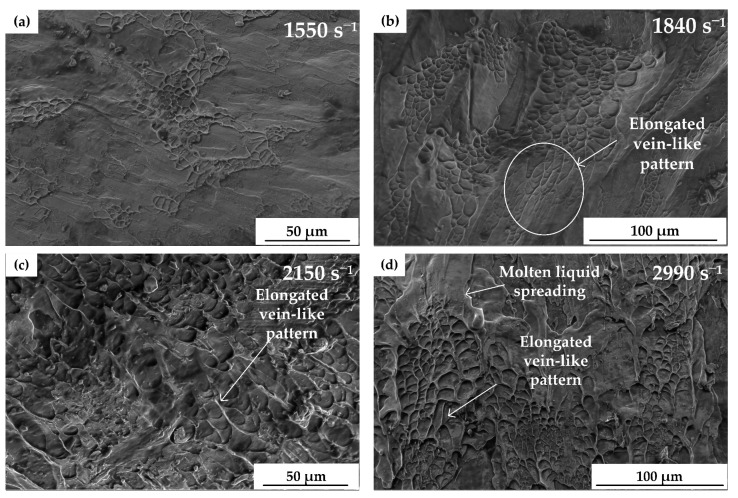
Vein-like pattern in the fracture surface of dynamic compressive tests. (**a**) 1550 s^−1^, (**b**) 1840 s^−1^, (**c**) 2150 s^−1^, and (**d**) 2990 s^−1^.

Figure 6 presents the fracture surface of (Cu_0.47_Zr_0.45_Al_0.08_)_98_Dy_2_ BMG at different strain rates in the dynamic tests. Vein-like patterns were distributed mainly along one direction on the fracture surface of the specimens of the quasi-static compressive tests, whereas those of the specimen of the dynamic compressive tests were distributed without clear direction, which indicated that the specimen was mainly subjected to axial compressive stress during quasi-static compressive, and in dynamic compressive tests, the specimen was subjected to both axial compressive stress and radial tensile stress. This is consistent with the analysis in Section 3.2.

When the strain rate was higher than 1840 s^−1^, elongated vein-like patterns were observed on the fracture surface. Within the strain rate range in this research, the higher the strain rate, the longer the vein-like pattern could be observed. The reason for this could be that the higher the loading energy of the dynamic test, a higher the adiabatic temperature rise in the shear bands, and the viscosity decreases faster, resulting in longer vein-like patterns [38]. A large area of molten liquid spreading was observed at a strain rate of 2990 s^−1^, indicating a higher temperature rise in the shear bands [20].

**Figure 7:**
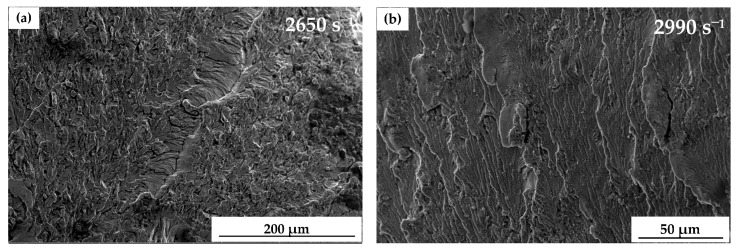
Rough region in the fracture surface of dynamic compressive tests: (**a**) 2650 and (**b**) 2990 s^−1^.

Under strain rates of 2650 and 2990 s^−1^, rough regions were observed in the fracture surface, as shown in Figure 7. The reason for this could be that the radial tensile stress caused by Poisson’s effect obviously affected the fracture of the specimen, the dynamic cracks were dynamically unstable during the rapid propagation [39,40]. It indicated that fracture occurred in both the shear mode and normal mode. The change in fracture mode could be the reason for the increase in yield stress at strain rates of 2650 and 2990 s^−1^.

## 4. Conclusions

This study carried out the quasi-static and dynamic compressive tests of (Cu_0.47_Zr_0.45_Al_0.08_)_98_Dy_2_ BMG at room temperature. The fracture morphology of static and dynamic specimens was analyzed by SEM. The following conclusions were drawn:

1. In the quasi-static compressive tests, the yield stress of (Cu_0.47_Zr_0.45_Al_0.08_)_98_Dy_2_ BMG increased from 1234 MPa to 1844 MPa when the strain rate increased from 0.001 s^−1^ to 0.01 s^−1^, and the yield stress decreased to 1430 MPa at the strain rate of 0.1 s^−1^.
2. In the dynamic compressive tests, when the strain rate increased from 1550 s^−1^ to 2990 s^−1^, the yield stress of (Cu_0.47_Zr_0.45_Al_0.08_)_98_Dy_2_ BMG first decreased from 1508 MPa to 1404 MPa, and then increased to 1593 MPa.
3. In the quasi-static compressive tests, the fracture exhibited a typical compressive shear fracture: a vein-like pattern, and vein-like patterns were distributed mainly along one direction, which indicated that the fracture occurred in the pure shear mode.
4. In the dynamic compressive tests, vein-like patterns were also observed on the fracture surface, and vein-like patterns were distributed without clear direction, and within the strain rate range in this research, the higher the strain rate, the longer the vein-like pattern could be observed. Under strain rates of 2650 s^−1^ and 2990 s^−1^, rough regions were observed in the fracture surface. The fracture surfaces indicated that fracture occurred in the pure shear mode with strain rates below 2100 s^−1^, whereas shear fracture and normal fracture occurred simultaneously under strain rates of 2650 s^−1^ and 2990 s^−1^.
